# Exploring barriers and facilitators, and their effectiveness in eye health promotion interventions: Protocol of a systematic review

**DOI:** 10.1371/journal.pone.0305904

**Published:** 2024-09-26

**Authors:** Hlabje Carel Masemola, Sphamandla Josias Nkambule, Olivia Baloyi, Zamadonda Nokuthula Xulu-Kasaba

**Affiliations:** 1 Department of Public Health Medicine, School of Nursing and Public Health, University of KwaZulu-Natal, Durban, South Africa; 2 Department of Optometry, College of Health Sciences, University of KwaZulu-Natal, Durban, South Africa; University of the Witwatersrand Johannesburg, SOUTH AFRICA

## Abstract

**Background:**

The rising global prevalence of visual impairment attributed to population growth and ageing heightens the risk of increased vision problems for many. However, there is a limited understanding of factors influencing intervention implementation, and the degree to which participation is achieved in interventions for eye health promotion by eye care professionals is currently limited.

**Aim:**

The purpose of this protocol is to review evidence on barriers and facilitators in eye health promotion, and their effectiveness on interventions employed in eye health promotion.

**Methods and expected outputs:**

The Center for Reviews and Dissemination (CRD) guidance for conducting systematic reviews in healthcare and reporting in accordance with the PRISMA statement will serve as the basis for the implementation of the proposed systematic review. Systematic literature searches will be performed by searching three databases, as well as hand-searching articles eligible for inclusion. The searches will include peer-reviewed articles presenting evidence on eye health promotion intervention from the inception of the Vision 2020 Campaign to the present day. RevMan 5.3 software will be used to gather quantitative data, such as (Effectiveness), meta-analysis of such data will be performed. Two reviewers will conduct a quality appraisal of the included articles using the mixed methods appraisal tool (MMAT) 2018 version. We expect to find relevant studies on health promotion interventions among health professionals at different levels of care. The synthesized evidence will help guide further implementation of research on health promotion interventions for eye health.

## 1. Background

Health promotion refers to interventions that are intended to improve population health and prevent illness [1]. Health promotion interventions are based on a variety of theories and models, including the socio-ecological model and the Health Belief Model. These models and theories focus on individual behaviours, community surroundings, and social structures [2]. These interventions aim to promote healthier lives, lower health risks, and address disparities through a variety of strategies, such as health education, policy changes, and community mobilization [3]. The literature presents evidence that health promotion interventions are effective in promoting positive health outcomes across a spectrum of health issues, such as infectious infections, mental health conditions, and chronic diseases [4, 5]. Furthermore, to design and implement interventions that are responsive to the needs and living conditions of diverse populations and ultimately improve population health and well-being, the field emphasizes the significance of taking a holistic approach that takes into account social determinants of health, cultural contexts, and equity principles [6, 7].

Enabling people and communities to take more control over their health and its determinants is crucial for health promotion, as defined by the Ottawa Charter for Health Promotion which contributes to achieving a state of optimal well-being [7]. The Ottawa Charter, which is based on the principles of empowerment, equity, and community involvement, offers a framework for holistically tackling health-related issues, including those of the promotion of eye health [8]. The Ottawa Charter’s action areas can be used to categorize interventions meant to improve eye health, including building healthy public policy, creating supportive environments, strengthening community action, developing personal skills, and reorienting health services [6]. Alignment of eye health promotion interventions with these action areas, this study seeks not only to review the nature and effectiveness of existing interventions but also to propose integrated strategies that address the needs of individuals at the community level.

Promoting eye health is an essential component of public health initiatives aimed to reduce the impact of vision impairment and improve overall community well-being [9, 10]. Major global public health issues include loss of vision and age-related eye conditions [11]. In addition to reducing an individual’s quality of life, vision impairment poses major economic and social challenges for communities [12]. Globally, at least 2.2 billion people have vision impairment, whereas at least 1 billion could have been prevented through health promotion actions [9]. In recent evidence, an improvement of 61% to 70% was observed in the uptake of eye care services following public ocular health campaigns as one of their eye care interventions [13]. In developing effective health interventions, it has become imperative for health professionals to play a crucial role in their execution and implementation [14]. This is because health professionals are responsible for the improvement of access and provision of quality health care to a target population [14]. Similarly, eye care professionals such as optometrists have been reported to provide health care services based on one of the core principles of the Ottawa Charter which is a global health milestone on health promotion; reorientation of services in the form of identification, evaluation, and prevention of disease and disorders [15]. Health promotion services, rehabilitation services, and health system management services also fall within these core principles [15]. Sithole(2022), affirms eye health promotion as an integral part of any action plan that seeks to address causative factors of various forms of visual impairment and blindness [16]. Primary healthcare services are excellent entry points for providing these interventions that promote health and reduce the risk of visual impairment [17]. Primary health care enables health systems to address the individuals’ health requirements, from health promotion to disease prevention, treatment, rehabilitation, palliative care, and more, as more people seek health care services, such as eye care [17]. This approach also guarantees that the delivery of healthcare is centred on patients’ needs and respects their preferences [18]. Effective health treatments have proved beneficial in preventing non-communicable diseases, which may be attributed to how they are delivered in different healthcare settings [19, 20]. When addressing these contexts, including implementation challenges and facilitators, it might be important to understand the techniques that improve the implementation of health-promoting interventions in various healthcare settings. According to the social-ecological model which focuses on multiple factors that might affect health, interactions between an individual, a group or community, and the physical, social, and political environments have an impact on one’s health. Such frameworks enhance the generalizability and interpretability of research findings in addition to increasing research efficiency [21].

Addressing the complex nature of eye health requires a comprehensive understanding of effective interventions, their implementation, and their impact on various populations [22]. Despite the existence of numerous interventions and strategies for eye health promotion which improve the uptake of eye care services, there is limited evidence on a comprehensive synthesis and evaluation of their effectiveness across diverse populations and settings [10]. Research that is currently available often focuses on specific interventions or particular populations, which could limit the generalizability of findings and hinder the establishment of broad approaches for the promotion of eye health [23]. This systematic review aims to address this gap by synthesizing evidence on a wide range of interventions for eye health promotion, including but not limited to preventive measures, educational programs, community outreach initiatives, and healthcare service delivery models [24]. Acknowledging the evidence will guarantee that interventions are customized to the contextual, socioeconomic, and cultural aspects affecting the health outcomes and behaviours related to eye health [25].

## 2. Methods

### Study design

This systematic review protocol is part of a larger study, “Developing an eye health promotion intervention framework within a primary health care setting in Limpopo Province, South Africa: A Mixed-Method Study”. This systematic review protocol was designed following the Preferred Reporting Items for Systematic Reviews and Meta-Analyses Protocol Extension (PRISMA-P) The PRISMA-P checklist is included S1 Table [26]. The protocol is registered with the International Prospective Register of Systematic Reviews (PROSPERO; CRD42022354299) https://www.crd.york.ac.uk/prospero/display_record.php?ID=CRD42022354299.

The methodology for the proposed systematic review will adhere to the guidelines for conducting systematic reviews in healthcare provided by the Centre for Reviews and Dissemination (CRD) [27]. In addition, a meta-analysis conforming to the standards of the Meta-analysis of Observational Studies in Epidemiology (MOOSE) will be carried out provided adequate data are available and studies are similar to interventions and outcomes [28]. This will be reported following the PRISMA 2020 statement: an updated procedure for reporting systematic reviews to ensure all necessary seven steps have been followed.

#### Step 1: Identifying the review question

Participants were not involved in the design and development of the study protocol. However, iterative consultation with key informants was conducted to define the research questions. Thus, to determine the research question’s eligibility for a systematic review project, the review question was guided by the Centre for Reviews and Dissemination (CRD) guidance for conducting systematic reviews in healthcare framed in terms of the population, intervention(s), comparator(s) and outcomes of the studies to be included [27].

The systematic review will address the following research questions;

1. What are the barriers and facilitators in the implementation of eye health promotion interventions?
2. What is the nature and effectiveness of interventions for eye health promotion?

#### Step 2: Eligibility criteria for considering studies for the systematic review

The eligibility criteria for considering studies for inclusion in this review are developed according to the relevant elements of the PICOd-T (Study Design—Time) framework in line with the research question [29]. The PICOd-T framework will be followed to ensure that the boundaries of the proposed systematic review research question are clearly defined [29]. Thus, eligible studies will be included after two reviewers have independently, reproducibly, and systematically evaluated them. Studies should present evidence on either of the factors, as illustrated in the S2 Table.

Participants will include studies among healthcare professionals residing in any part of the world. Interventions will consist of any interventions or a combination of interventions aimed at influencing the timeliness of symptomatic eye health diagnosis and screening asymptomatic individuals along any or all of the five key areas/ core focus of the Ottawa Charter framework for health promotion, whether as a single-focus intervention or a multi-focus intervention targeting multiple eye health services. The outcome will involve any clinical outcome, including (but not restricted to) improved access to eye health care, behavioural change, prevention of blindness, improved visual acuity and evidence-based policy and legislative change. The review will include primary studies from randomised controlled trials, quasi-experimental, observational and qualitative studies that are peer-reviewed. Research studies reporting on any other interventions that do not promote eye health will be excluded. The standard of care will represent the comparator. Outcomes of interest will include any or all of the outcomes or target goals of the Ottawa Charter framework for health promotion, such as improved access to early diagnostic services, reduced diagnostic time, and others—authors of studies published only as abstracts will be contacted and asked to provide further details. If no further detail is available, the source will be excluded. A third reviewer will resolve any disagreements between the two reviewers; furthermore, two reviewers will test the inclusion/exclusion criteria through piloting to establish agreement before commencing the study selection process.

#### Step 3: Search strategy for the identification of studies addressing the review question

Systematic, comprehensive, and reproducible searches of reputable bibliographic databases, and indexing services (and platforms), followed by other supplementary information sources, will be conducted to capture primary studies addressing the main review question. Two reviewers will perform all the direct electronic and supplementary systematic searches using a pre-defined and piloted search strategy with the assistance of a professional librarian. Both electronic bibliographic database searching and additional search techniques will be covered by the search strategy to find published and unpublished (grey) literature that will be screened for inclusion in this study. The World Health Organization package will inform our search strategy of eye care interventions. A preliminary search for existing systemic reviews on this topic under review was conducted on 20 November 2023 in PubMed, EBSCOhost platform and Web of Science. The initial search strategy was developed in PubMed with 17,804 references identified. The search will be conducted in English and the authors acknowledge that literature in another language will be missed. The manuscript will recognize implicit constraints by referencing relevant literature, such as the study conducted by Amano et al,(2016) which suggests that relying solely on English-language sources may result in overlooking approximately 35% of relevant literature [30].

The first author, a librarian and a subject specialist, co-developed the comprehensive search strategy S3 Table. All authors will be given an equal opportunity to review the draft to ensure the correct use of indexing terminology and Medical Subject Headings (MeSH) descriptors before pilot testing on a subset of records from the Web of Science database. The details of the search strategy metrics–descriptors and truncation used, as well as the number of returned pilot records, are presented in S3 Table–this primary search strategy will then be adapted for other databases. A search summary table (SST) will be used to report on the search strategy methods and their effectiveness S5 Table.

### Electronic search sources

Advanced systematic searches employing the piloted search strategy will be conducted from the following electronic databases to source articles published from the inception of the Vision 2020 campaign in 1999 to November 2023: WEB of Science, PubMed, and EBSCOHost Web (Academic Search Complete, CINAHL Complete, MEDLINE with Full Text, CINAHL with Full Text and Health Source: (Nursing/Academic Edition). Furthermore, randomized controlled trials that report evidence on health promotion interventions will be sourced from the South African National Clinical Trial Register and ISRCTN registry.

All three electronic databases (PubMed, EBSCOhost platform and Web of Science) will be searched during the search strategy according to the criteria. This process aims to obtain the maximum number of sources from the electronic databases and ensure that all relevant articles or reports are captured before the study selection and eligibility screening process begins.

### Searching other resources–supplementary information search

In addition, supplementary search methods will include hand-searching of relevant journals, reference lists of identified peer-reviewed articles and grey literature, and as well as forward and backwards citation chasing. The reviewers will further browse through the link entitled "Related Articles" option, which searches for similar citations using an intricate algorithm that scans titles, abstracts, and MeSH terms to detect more studies.

Systematic reviews and other review papers are not eligible for inclusion; however, reference lists of relevant reviews, preprints, and conference abstracts will be screened for more relevant primary studies not captured by the search strategy. Furthermore, the appropriate trial publications reference lists will be checked for unidentified randomized clinical trials.

### The systematic search management

The search summary table (SST) will be used to document the effectiveness of the search strategy, the systematic searches performed across databases, information used to inform the PRISMA-2020 flow diagram S2 Fig, and the search methods, as well as the additional information gathered by the librarian or information specialist in their search log. Using the SST format to present the information mentioned above will allow for calculating various search effectiveness metrics, and reporting these metrics shows the effectiveness of search strategies for each database and database searching as a whole. Furthermore, reporting these metrics separately increases the transparency of the search and study selection process.

The SST will be completed in two stages. In stage one, all the references that the search strategy retrieves from each electronic database, including all duplicates, will be exported to EndNote X9 (version 19.1.0.12691)–a reference management software, which will be used to create a virtual library (Thomson Reuters, Stamford, CT, USA). Every record in the virtual library will be given a code for the database name where the record was found. Stage two involves re-running the searches in the databases where most of the included references were found to determine whether references not found during the original search were in the database and, if so, whether the search strategy retrieved them.

#### Step 4: Study selection and eligibility screening

The procedure for study selection and screening for eligibility will be carried out according to the Preferred Reporting Items for Systematic Reviews and Meta-Analysis (PRISMA) 2020 guidelines in S2 Table. A PRISMA-2020 style flowchart will be produced detailing the study selection and screening process and the reason for excluding each full-text paper reported as illustrated in S2 Fig.

The study selection and screening procedure will be done in three folds: Title screening, abstract screening and full-article screening; each stage will be guided by data screening tools developed in Google format (see Supplementary Information for examples of the data screening tools). The abstracts and titles of references retrieved by the electronic searches will be screened independently by two reviewers using the pre-specified inclusion/exclusion criteria S1 Table. Discussions will resolve discrepancies with a third reviewer (JTC) if necessary.

The full text of potentially relevant studies will be obtained following the abstract and title screening stages. Furthermore, guided by the same methods, the full text of articles deemed eligible will be assessed for inclusion by two reviewers. Using Stata 13.0SE (Stata Corp College Station, TX, USA), the Cohen’s Kappa coefficient (κ) statistic will be used to assess the disagreements between the two reviewers. When measuring inter-rater reliability, Cohen’s Kappa coefficient (κ) statistic is a reliable statistical technique [31]. In order to settle disagreements that have been identified between the two reviewers, a third reviewer will be asked for input.

#### Step 5: Data extraction/ or collection process

The data extraction process will be informed by the template for intervention description and replication (TIDieR) checklist and guide for describing interventions S5 Fig. The overarching purpose of the TIDieR checklist is to prompt authors to describe interventions in sufficient detail to allow their replication. The checklist contains the minimum recommended items for describing an intervention. Thus, for this study and to ensure that the data collection process is reproducible, a standardised data extraction tool prepared in a Google Form format, based on the TIDieR checklist, will be used for extracting relevant data to characterize included studies (see Supplementary Information for examples of the data screening tools). Feedback was solicited from all authors regarding the draft list of data variables for extraction. The final data extraction tool will be calibrated and piloted for reliability and applicability on the first three eligible studies to maintain accuracy and ensure all necessary information is captured.

Quantitative data will be extracted by the first author/ or reviewer into a piloted, standardized data extraction form; the second reviewer will check this, and discrepancies will be resolved by discussion with the involvement of a third reviewer (JTC) if necessary. For qualitative studies, details of the study aim, the sample, and the type and nature of the intervention/programme, as well as data on the theoretical approach, the methods used to collect the data, the analytic processes and the quotes, themes and concepts pertinent to our research questions will be extracted into a piloted standardized data extraction form. Two reviewers working simultaneously and independently will conduct this process to exhibit strong inter-rater reliability. Any discrepancies will be resolved through discussion. A structured summary will be produced for each paper, and the extracted data will be tabulated, allowing comparison between studies S7 Table.

### Requests for missing data

Missing data will be identified and recorded within the review. Where means and standard deviations are not available, we will collect effect estimates, confidence intervals, test statistics, P values and individual participant data where reported. Where papers provide insufficient details about the intervention, such as what is delivered and by whom, an effort would be made to contact the authors to obtain previously unpublished information and clarify any missing data.

#### Step 6: Assessment of study methodological quality and risk of bias

The methodological quality and risk of bias of each included study will be assessed by two reviewers independently, using appropriate methodological quality assessment tools depending on the design of the included studies.

### Tools to assess the methodological quality

As recommended, the mixed-method appraisal tool (MMAT) version 2018 will be used to determine the methodological quality of the included studies [32]. The MMAT appraisal tool consists of two core assessment domains addressing the external validity (risk of selection, data collection and non-response bias) and five further assessment domains addressing the internal validity (systematic error—the risk of measurement bias and bias related to data analysis) deemed relevant to appraise the methodological quality of various studies. To generate an overall methodological quality assessment, a percentage quality score will be calculated for each included study based on the evaluation of each domain and the overall risk of bias across domains. The scores will be interpreted as low quality if ≤ 50%, average quality if 51–75%, and high quality if 76–100%.

### Tools to assess risk of bias (or study limitations)

The risk of bias in individual studies will be assessed using a suitable tool by one reviewer (VPN), checked by a second reviewer (MXR), and any disagreement will be resolved by discussion involving a third reviewer (HCM) if necessary. To determine the effectiveness and cost-effectiveness of interventions for eye health promotion, details on effect measures used to describe effect sizes in included studies and meta-analyses, such as risk ratio or odds ratio, mean difference or standardized mean difference, will be extracted. However, only measures most adjusted for one or more sets of potential confounders, such as socio-demographic and lifestyle factors, will be extracted to reduce confounding and measurement errors, ensure consistency across studies and reduce bias. The most appropriate measures for synthesis will be used, depending on the availability and appropriateness of available data, and document our decision-making processes.

### Tools to assess the certainty of evidence (quality of evidence)

The quality of quantitative evidence will be ascertained using the current Grading of Recommendations Assessment, Development and Evaluation (GRADE) guidelines for evaluating the certainty of a body of evidence [33]. The GRADE-CERQual will be used to establish the degree of confidence that may be placed in the qualitative evidence synthesis findings; and the results will be reported alongside the Summary of Findings table, together with a justification for judgement, in the ‘Included studies’ section. In the case of a CRT, a special variant of the RoB 2 tool focusing mainly on group participants from the clusters will be used [33].

#### Step 7: Collating, summarizing, and reporting the results

The proposed systematic review aims to integrate health promotion interventions in practice by health professionals within the framework of the Ottawa Charter and also identify barriers and facilitators in the implementation of eye health promotion interventions using a Social-Ecological framework. Initially, upon the completion of the data extraction phase, a summary of findings table created from the collected data from the included studies will be generated, presenting a descriptive numerical description of the data. The descriptive summary of the findings table will include each of the pre-specified outcomes S2 Table, and details of the characteristics of the included studies, such as the total number of publications included, type of study design, year of publication, type of risk factors reported, characteristics of the study populations, and study setting.

Secondly, a world map will be modelled using Canva version 2.93.0, an interactive web-based graphic design application, in order to display the patterns of variation in the incidence, distribution, and trends of the studies that were selected for review. Thirdly, tables and figures will be used to present the results in an organized way to satisfy the main research question addressed by the proposed systematic review.

We will collate the data using an NVivo version 12 to present a narrative account of the literature through content thematic analysis of the included studies. Excerpts of text will be coded deductively by the reviewers to identify concepts and themes related to the research questions. The resulting themes will be analysed and the result will inspect the relationship between the research questions and the findings. In this step of the systemic review, we will provide a summary of the evidence applying the five key action areas of the Ottawa Charter to chat the outcomes. The Social-Ecological model for health promotion will be used as a framework to synthesize and categorize facilitators and barriers influencing the implementation of eye health promotion interventions. The meaning of the findings will be considered as they relate to the overall study purpose and the implications of these findings for future research, policy, and practice.

## 3. Discussion

Our systemic review builds on reviewing literature addressing health promotion interventions practised by eye health professionals within the context of primary healthcare. This systemic review aims to collect evidence on the health promotion interventions practised by eye health professionals within the framework of the Ottawa Charter and also identify barriers and facilitators in the implementation of eye health promotion interventions using a Social-Ecological framework. It is anticipated that findings from this review identify gaps and guide future research to bridge the gaps toward improved health outcomes for individuals using primary eye care services. The results of this review will guide eye care services implementers in designing health promotion interventions that will help improve individuals’ engagement with primary eye care services in South Africa.

Health promotion is one of the strategies recommended by the WHO World Report on Vision to address eye care needs [9]. Health promotion interventions such as awareness and education are important aspects of an eye health action plan that seeks to address causative factors of various burdens of eye diseases such as cataracts, glaucoma, and blinding uncorrected refractive errors [16]. The burden of eye diseases has serious consequences on the social determinants of health which can be mitigated by appropriate access to quality eye care [9]. In sub-Saharan Africa, health care is mostly provided through the public health service [34]. District Health Service (DHS) consists of primary, secondary, and tertiary levels, which in most instances focus on providing curative care. DHS is associated with barriers to accessing eye care, limited engagement with communities, a shortage of appropriately skilled health personnel, and inadequate support from health systems [34]. Global action plans for the prevention of avoidable blindness and visual impairment recommend an increase in public awareness and use of eye healthcare services [35].

Our findings expect to have relevant studies on health promotion interventions by health professionals at different levels of care. Therefore, the evidence of this systemic review will guide eye health professionals in planning intervention strategies to address public health concerns in eye health. The findings will guide further implementation research on health education interventions for eye health. We will present this review at scientific conferences on health promotion and distribute it online or in print. To the best of our knowledge, no systemic review studies on health promotion interventions by health professionals have been conducted previously.

## 4. Strengths and limitations of the study

The strength of this study is that it will apply a broad review of multidisciplinary databases covering ophthalmology, medicine general internal, paediatrics, surgery, health care science services, public environmental occupational health, clinical neurology, endocrinology metabolism, multidisciplinary science, medicine research experimental and other Web of Science Core Collection Citation Topic. This approach will provide a comprehensive assessment of published literature on courses designed on these subjects as illustrated in S6 Fig.

This is a significant systemic review to synthesize qualitative and quantitative evidence on the nature and effectiveness of interventions for eye health promotion practised by health professionals within different levels of care. This review will broaden to inform study research for the development of a framework to guide the implementation of eye health interventions in South Africa. Health promotion practitioners and relevant stakeholders will be engaged throughout the study. A systemic review approach was chosen for this phenomenon as other reviews may suffer from publication bias. The review is also limited to studies published in English which may bias the evidence.

## Supporting information

S1 Fig PICO framework for determining the eligibility of the research question. (PDF)

S2 Fig PRISMA 2020 flow diagram outlining the study selection and eligibility. (PDF)

S3 Fig Title & abstract screening form. (PDF)

S4 Fig Full-text screening form. (PDF)

S5 Fig Distribution of publications selected from Web of Science core collection by categories. (PDF)

S6 Fig Distribution of publications selected from Web of Science core collection citation topic. (PDF)

S7 Fig Distributions publications (Topic). (PDF)

S1 Table PRISMA-P checklist. (PDF)

S2 Table Eligibility criteria (PICOd-T). (PDF)

S3 Table Search strategy. (PDF)

S4 Table Meta-analysis data capturing sheet. (PDF)

S5 Table Search summary. (PDF)

S6 Table Categories of themes and subthemes of interest. (PDF)

S7 Table Data extraction. (PDF)
