# Supplementary figures and images for: Exploring barriers and facilitators, and their effectiveness in eye health promotion interventions: Protocol of a systematic review
Source: PLoS One. 2024 Sep 26;19(9):e0305904. doi: 10.1371/journal.pone.0305904 (PMC11426475; doi:10.1371/journal.pone.0305904)

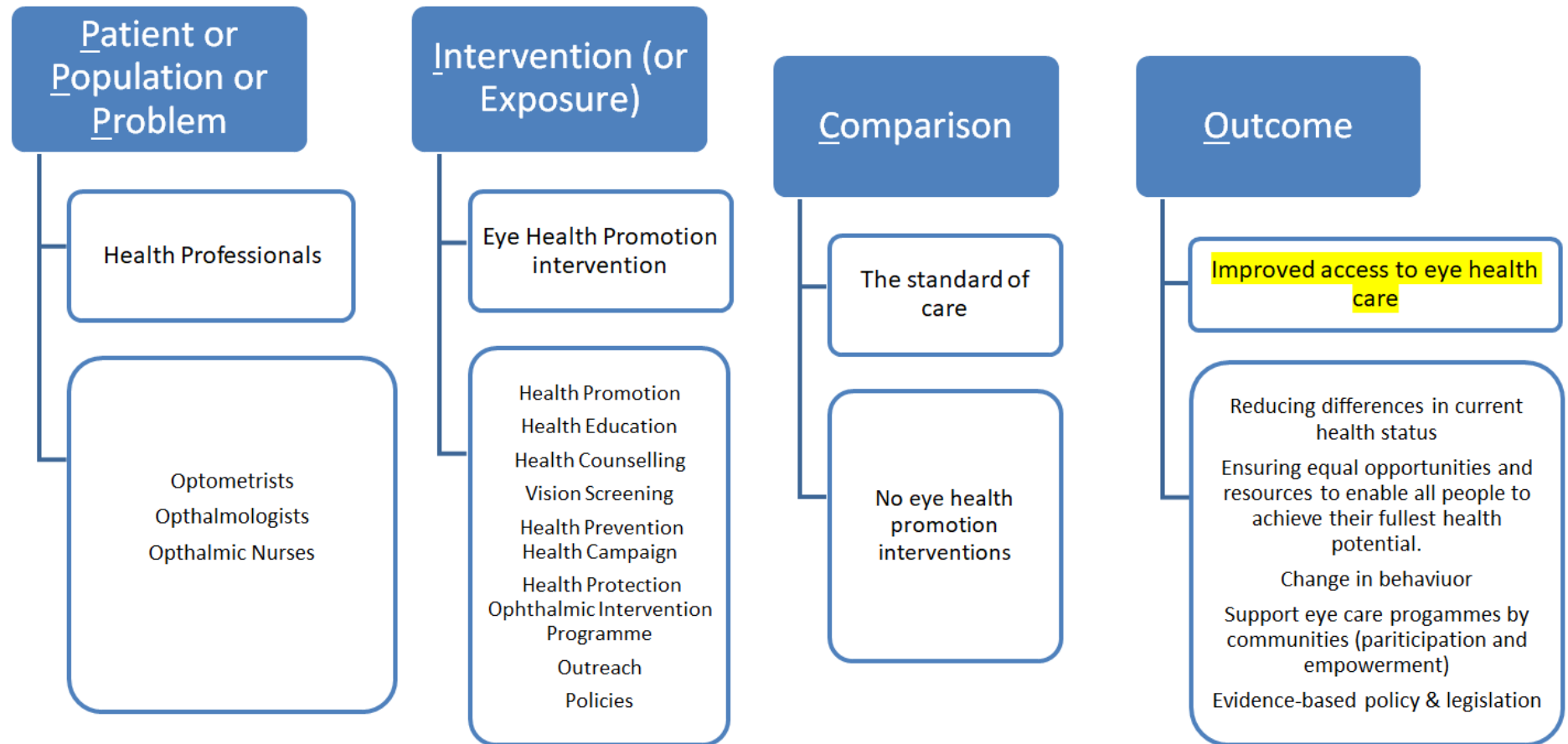

Supplement: S1 Fig — (PDF) [file pone.0305904.s001.pdf]

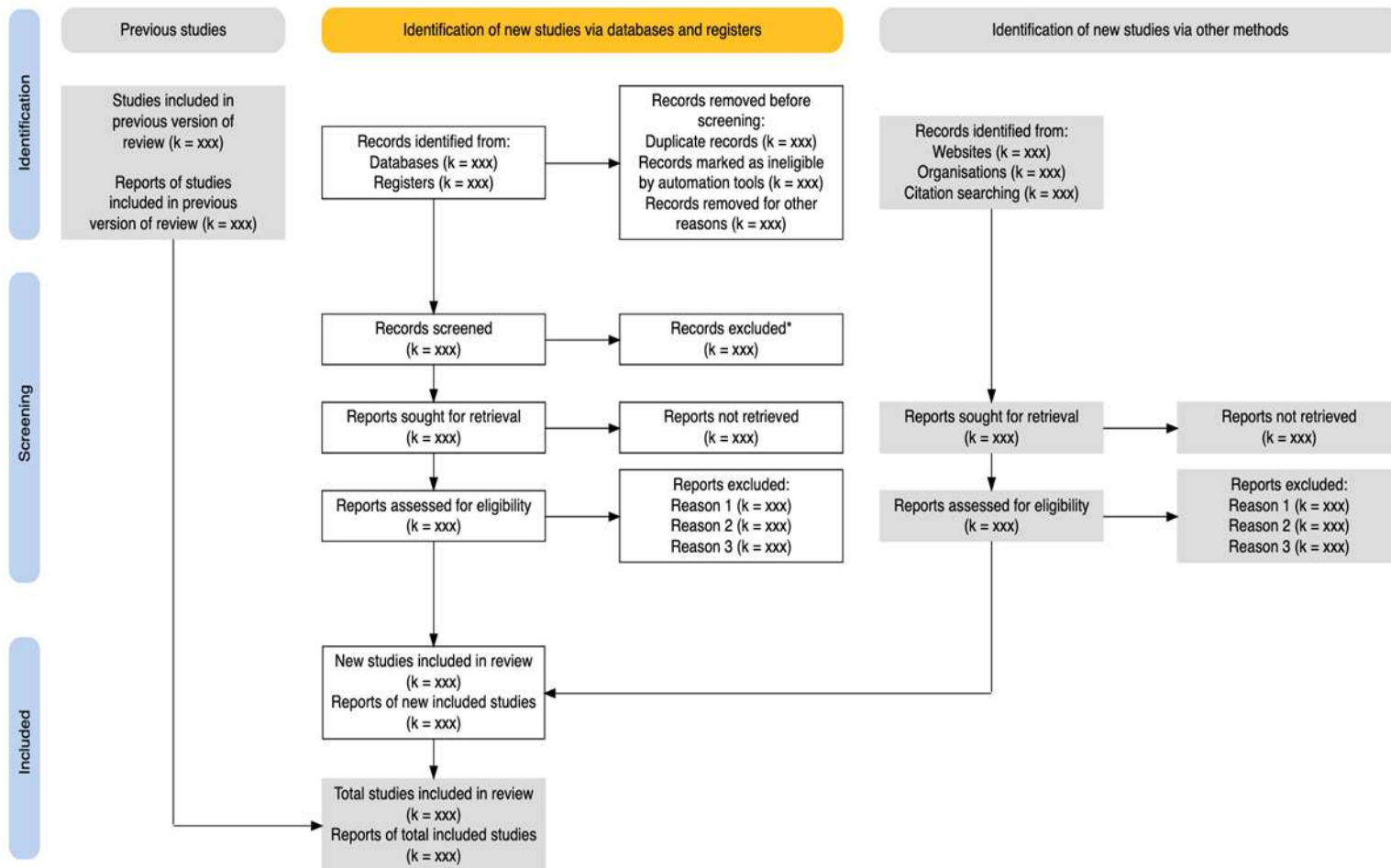

Supplement: S2 Fig — (PDF) [file pone.0305904.s002.pdf]

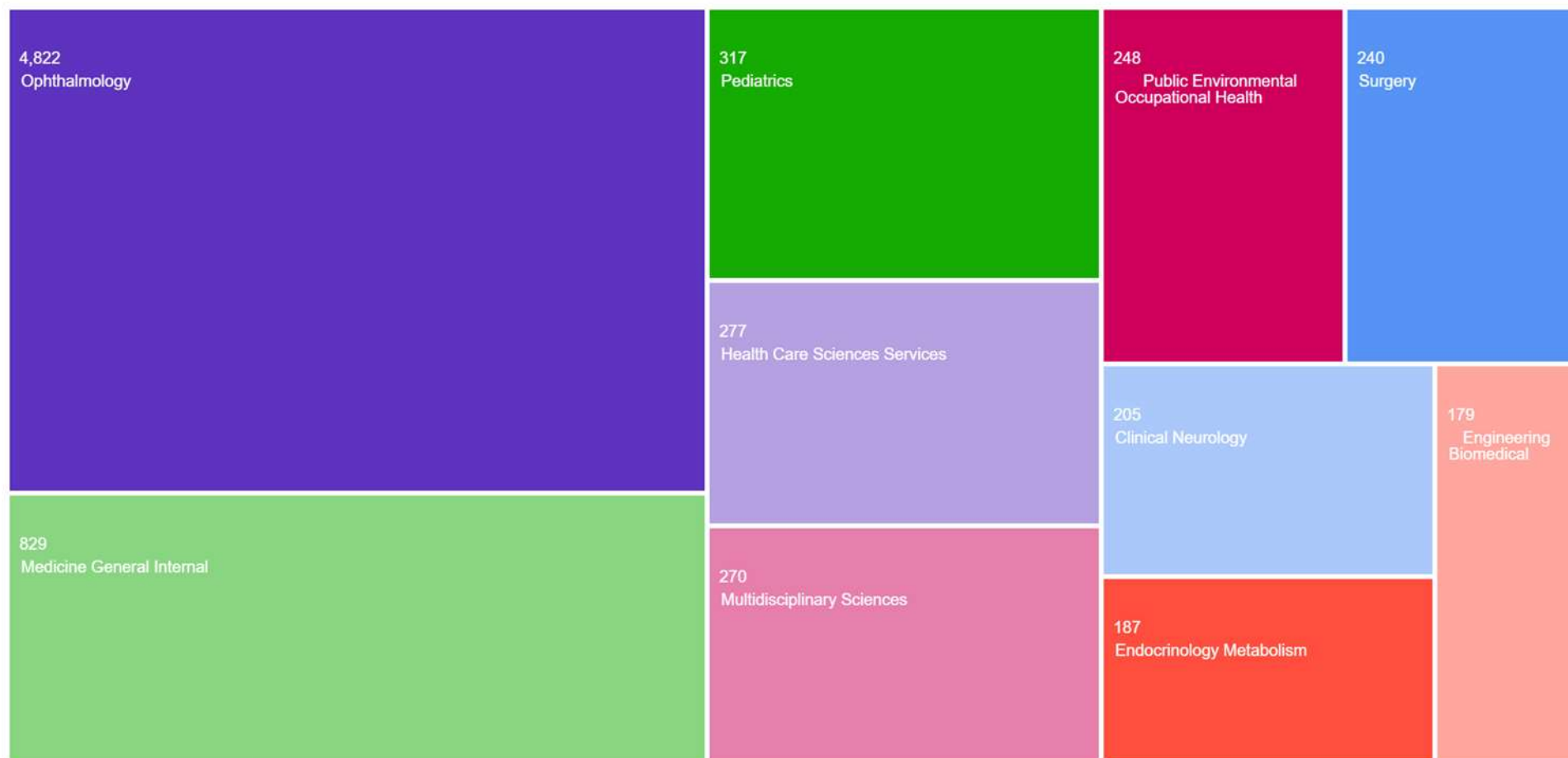

Supplement: S6 Fig — (PDF) [file pone.0305904.s006.pdf]

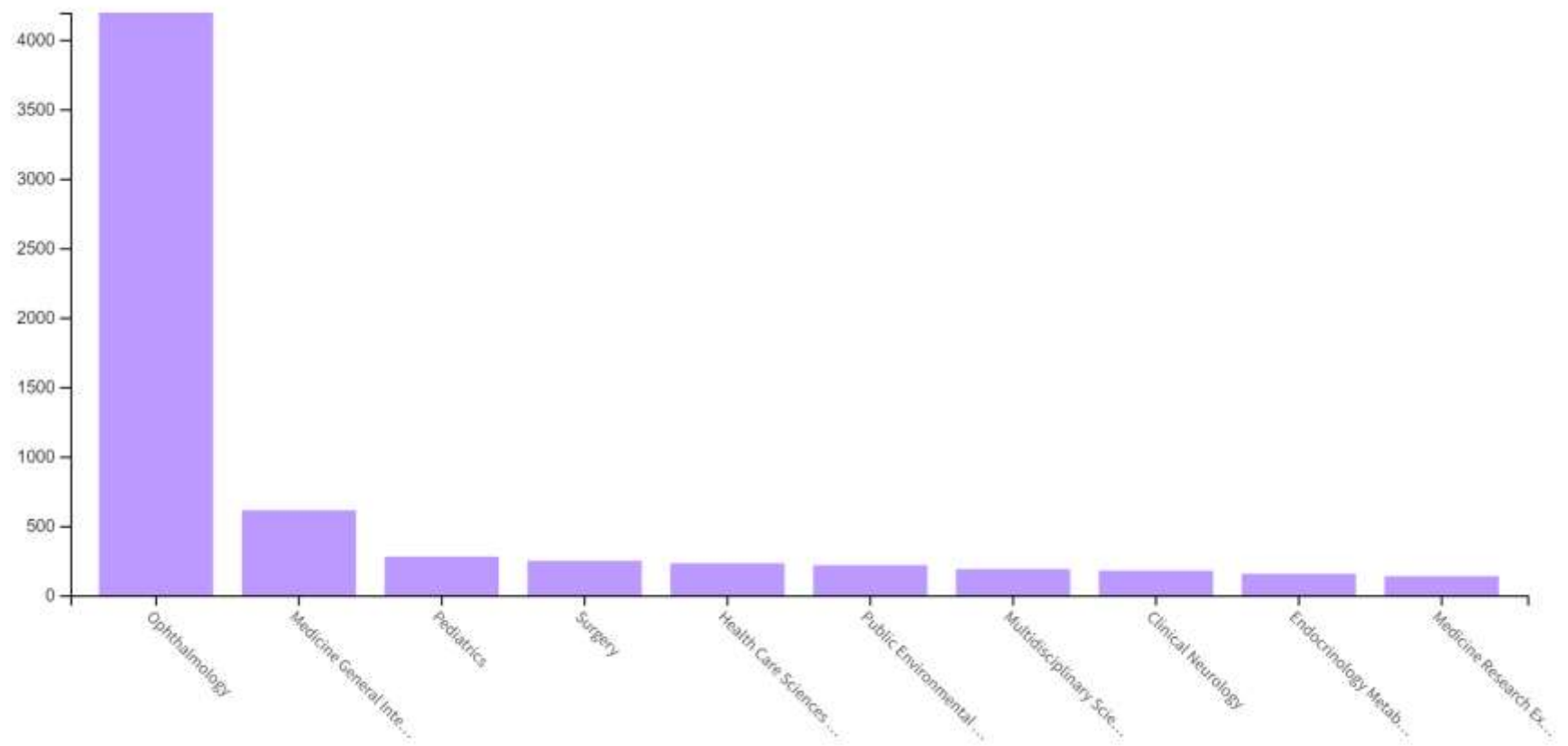

Supplement: S7 Fig — (PDF) [file pone.0305904.s007.pdf]
