# Supplementary material for: Exploring barriers and facilitators, and their effectiveness in eye health promotion interventions: Protocol of a systematic review
Source: PLoS One. 2024 Sep 26;19(9):e0305904. doi: 10.1371/journal.pone.0305904 (PMC11426475; doi:10.1371/journal.pone.0305904)
Supplement: S3 Fig — (PDF) [file pone.0305904.s003.pdf]

Exploring Barriers and Facilitators in Eye Health Promotion Interventions: A Systematic Scoping Review

Title & Abstract - Level 1 Screening Form

(Preliminary One)

* Indicates required question

1. **Author, Year**

2. **Title** *

3. **What is the Aim/ or Objectives of this research study?** *

4. **Does this research study present evidence [*implementation*] of an intervention for eye health promotion?** *

Mark only one oval.

- Yes
- No

5. **Does this research study present evidence on the *nature and effectiveness* of interventions for eye health promotion?** *

Mark only one oval.

- Yes
- No

6. **Who is the Screen-er?** *

Mark only one oval.

- Xolani
- Portia

This content is neither created nor endorsed by Google.

Google Forms
