# Supplementary material for: Exploring barriers and facilitators, and their effectiveness in eye health promotion interventions: Protocol of a systematic review
Source: PLoS One. 2024 Sep 26;19(9):e0305904. doi: 10.1371/journal.pone.0305904 (PMC11426475; doi:10.1371/journal.pone.0305904)
Supplement: S4 Fig — (PDF) [file pone.0305904.s004.pdf]

Exploring Barriers and Facilitators in Eye Health Promotion Interventions: A Systematic Scoping Review

Full-Text - Level 2 Screening Form (Preliminary Two)

* Indicates required question

1. **Author, Year** *

2. **Title** *

3. **What is the Aim/ or Objectives of this research study?** *

4. **Country of Study** *

5. **Intervention Settings** *

Mark only one oval.

- Hospital based
- School based
- Clinic based
- Community based
- Others
- Not specified

6. This research study was conducted in which country according to the ***World Bank country income classifications***? *

Updated country income classifications are available here.

Mark only one oval.

- LOW-INCOME ECONOMIES ($1,085 OR LESS)
- LOWER-MIDDLE INCOME ECONOMIES ($1,086 TO $4,255)
- UPPER-MIDDLE-INCOME ECONOMIES ($4,256 TO $13,205)
- HIGH-INCOME ECONOMIES ($13,205 OR MORE)
- Not Clear

7. **Does this research study present evidence on eye health intervention conducted by eye health professionals?** *

Mark only one oval.

- Yes
- No

8. **Does this research study present evidence on the implementation of the intervention for eye health promotion?** *

Mark only one oval.

- Yes
- No

9. **Eye health professional involved in the intervention** *

Check all that apply.

- [ ] Optometrist
- [ ] Ophthalmologist
- [ ] Ophthalmic Nurse

10. **Which type of eye health promotion is reported?** *

Mark only one oval.

- Health Promotion
- Health Education
- Health Counselling
- Health Prevention
- Vision Screening
- Outreach
- Policies
- Ophthalmic Intervention Programme
- Behavioural Change

11. **Description of the Intervention** *

Include the duration of program

12. **Study participants total (n=?)** *

13. **Response rate?** *

14. **Intervention settings/ Site, Country of Study** *

Examples: Hospital-Based Healthcare, University, Community-Based Healthcare, and School -Based

15. **Study Design**

Examples: Retrospective Pretest/ Post Test Survey, Structured interviews, Mixed Methods

16. **Analysis used?** *

17. **Tool(s) of measure** *

18. **Pre/ Post- Intervention Outcomes (*), and effectiveness** *

(*) indicates statistically significant result, approaches used to evaluate the program outcomes

19. **Authors Recommendations** *

Example: Future Research and Practice

20. **Barriers/ Challenges encountered in the design and implementation of the program** *

21. **Authors Conclusions** *

Research study key findings

22. **Study Team Notes** *

All systematic and random error adjusted, confounding, adjusted covariates, effect medication etc.

23. Does this research study present evidence on the **nature** and **effectiveness** of interventions for eye health promotion? *

Mark only one oval.

- Yes
- No

24. Does this research study present evidence on any outcome related to improved eye health care services? *

Mark only one oval.

- Yes
- No

25. Does this research study present evidence on factors that serve as facilitators and barriers to interventions for eye health promotion? *

Mark only one oval.

- Yes
- No

26. Who is the Screen-er? *

Mark only one oval.

- 1st Reviewer (**)
- 2nd Reviewer (**)

This content is neither created nor endorsed by Google.

Google Forms
