# Supplementary material for: Exploring barriers and facilitators, and their effectiveness in eye health promotion interventions: Protocol of a systematic review
Source: PLoS One. 2024 Sep 26;19(9):e0305904. doi: 10.1371/journal.pone.0305904 (PMC11426475; doi:10.1371/journal.pone.0305904)
Supplement: S5 Fig — (PDF) [file pone.0305904.s005.pdf]

Exploring Barriers and Facilitators in Eye Health Promotion Interventions: A Systematic Scoping Review

Data Extraction - Level 3 Screening Form (Preliminary Three)

* Indicates required question

1. **Author, Year** *

2. **Title** *

3. **What is the Aim/ or Objectives of this research study?** *

4. **Country of Study** *

5. **Intervention Settings** *

Mark only one oval.

- Hospital based
- School based
- Clinic based
- Community based
- Others
- Not specified

6. This research study was conducted in which country according to the ***World Bank country income classifications***? *

Updated country income classifications are available here.

Mark only one oval.

- LOW-INCOME ECONOMIES ($1,085 OR LESS)
- LOWER-MIDDLE INCOME ECONOMIES ($1,086 TO $4,255)
- UPPER-MIDDLE-INCOME ECONOMIES ($4,256 TO $13,205)
- HIGH-INCOME ECONOMIES ($13,205 OR MORE)
- Not Clear

7. **Does this research study present evidence on the implementation of the intervention for eye health promotion?** *

Mark only one oval.

- Yes
- No

8. **Eye health professional involved in the intervention** *

Check all that apply.

- [ ] Optometrist
- [ ] Ophthalmologist
- [ ] Ophthalmic Nurse
- [ ] Ophthalmic Technicians
- [ ] Not Specified

9. **Which type of eye health promotion is reported?** *

Mark only one oval.

- ( ) Health Promotion
- ( ) Health Education
- ( ) Health Counselling
- ( ) Health Prevention
- ( ) Vision Screening
- ( ) Outreach
- ( ) Policies
- ( ) Ophthalmic Intervention Programme
- ( ) Behavioural Change

10. **Description of the intervention**
Include the duration of the intervention

____
____
____
____
____

11. **Study design** *

____

12. **Study participants total (n=?)** *

13. **Response rate?** *

14. **Analysis used?** *

15. Tool(s) of measure *

16. **Level of the Socio-ecological model** *

Check all that apply.

- [ ] Intrapersonal
- [ ] Interpersonal
- [ ] Organisational
- [ ] Environmental/Community
- [ ] Public policy

17. **Action areas of the Ottawa Charter for health promotion** *

Check all that apply.

- [ ] Build Healthy Public Policy
- [ ] Create Supportive Environments
- [ ] Strengthen Community Actions
- [ ] Develop Personal Skills
- [ ] Reorient Health Services

18. **Pre/ Post- Intervention Outcomes (*), and effectiveness** *

(*) indicates statistically significant result, approaches used to evaluate the program outcomes

19. **Authors Recommendations** *

Example: Future Research and Practice

20. **Barriers/ Challenges encountered in the design and implementation of the program** *

21. **Authors Conclusions** *

Research study key findings

22. **Study Team Notes** *

All systematic and random error adjusted, confounding, adjusted covariates, effect medication etc.

23. Who is the Screen-er? *

Mark only one oval.

- 1st Reviewer (**)
- 2nd Reviewer (**)

This content is neither created nor endorsed by Google.

Google Forms
