# Supplementary material for: Exploring barriers and facilitators, and their effectiveness in eye health promotion interventions: Protocol of a systematic review
Source: PLoS One. 2024 Sep 26;19(9):e0305904. doi: 10.1371/journal.pone.0305904 (PMC11426475; doi:10.1371/journal.pone.0305904)
Supplement: S2 Table — (PDF) [file pone.0305904.s009.pdf]

Criteria	Inclusions	Exclusions
**Population**	Studies conducted in any part of the world with human participants, general public or health professionals, irrespective of study setting, will be eligible for inclusion.<br><br>Reported study participants will be included irrespective of gender, ethnicity, culture, race, and comorbidities background.	Research studies involving non-human participants as participants.
**Intervention (or Exposure Risk Factor of interest)**	Studies with human participants exposed to interventions that consist of strategies aimed at influencing the timeliness of symptomatic eye health diagnosis along any or all of the five key areas of the Ottawa charter framework for health promotion.<br><br>Studies will be included either reporting on interventions, whether as a single focus intervention or a multi-focus intervention targeting multiple eye health conditions regardless of exposure history/ period post-diagnosis:<br>• Examples = Health education, community outreach, school health services, promotion of eye awareness, eye health promoting behaviours, policies that promote eye health, literacy programme, incorporation of community members in eye care service delivery, nutrition programmes, accessibility to sanitation and safe drinkable water relevant to the prevention of trachoma, integration of eye care services, increased focus on research into eye conditions, eye health advocacy, expansion of eye care services, mass treatment, vector control, provision of spectacles, eye safety legislation, food policy and fortification,	Research studies reporting on any other interventions of not eye health promotion.<br><br>Studies which do not adequately describe the intervention will be excluded.
**Comparator**	The standard of care will represent the comparator. Studies reporting on outcomes of interests, interventions support with no support/ or standard of care or compare one type of interventions support with another<br>Examples = effective; safe; people-centred; timely; equitable;	

Criteria	Inclusions	Exclusions
	integrated; and efficient. (Any active or inactive comparator)	
**Outcomes**	Studies reporting on the following outcomes of interest, including any or all of the outcomes or target goals of the Ottawa charter framework for health promotion, such as improved access to early diagnostic services, reduced diagnostic time, and Other eye health services<br>Examples = Improved access to eye health care, reducing differences in current health status, ensuring equal opportunities and resources to enable all people to achieve their fullest health potential, Change in behaviour, support eye care programmes by communities	Research studies reporting on other outcomes not in line with the Ottawa charter framework for health promotion.
**Study Design**	Only original, primary studies of both quantitative and/ or qualitative study designs that are peer-reviewed and published in English, irrespective of publication year will be eligible for inclusion<br>Examples=	Systematic reviews (However, reference lists of relevant reviews will be checked for primary studies)<br>Case reports, Anecdotal reports, Modelling studies, and unpublished/ or grey literature will not be included in this review.<br>Authors of studies published only as abstracts will be contacted and asked to provide further detail. If no further detail is available, the study will be excluded.
**Time**	From 2009 – up to November 2023	
**Language**	Studies written in English will be included to avoid loss or distortion by translation from studies written in another language.	Non-English language quantitative and qualitative studies will be excluded.

Criteria	Inclusions	Exclusions
**Note:** The inclusion/exclusion criteria will be tested through piloting by two reviewers to establish agreement prior to commencing the study selection process.		
