# Supplementary material for: Exploring barriers and facilitators, and their effectiveness in eye health promotion interventions: Protocol of a systematic review
Source: PLoS One. 2024 Sep 26;19(9):e0305904. doi: 10.1371/journal.pone.0305904 (PMC11426475; doi:10.1371/journal.pone.0305904)
Supplement: S3 Table — (PDF) [file pone.0305904.s010.pdf]

	Population	Intervention	Comparators	Outcomes
**Key concepts**	**Eye Health Professionals**	**Eye Health Promotion**	**Standard of care/ Usual Care**	**Prevention of Blindness**
**Free text terms / natural language terms**<br><br>(synonyms, UK/US terminology, medical/laymen's terms, acronyms/abbreviations, drug brands, more narrow search terms)	Optometrist OR Ophthalmologist OR Ophthalmic Nurse OR Ophthalmic Practitioner OR Ophthalmic Assistant OR Ophthalmic technician OR Eye care professional OR Eyecare professional	Health OR Vision OR Ocular OR Eye care OR Eye care OR Eye disease OR Intervention OR Outreach School health service OR Advocacy OR Service improvement or Program OR Promotion OR Education OR Counselling OR Screening OR Campaign	As reported in the study	Reduce ocular morbidity<br><br>Increase the use of eye care services<br><br>Improving eye health
**Controlled vocabulary terms / Subject terms**<br><br>(MeSH terms, Emtree terms)	Search: (((Optometrists) OR (Ophthalmologists)) OR (Ophthalmic Nurses)) OR (Ophthalmic Practitioner)	Search: ((((((((((Health Promotion) OR (Health Education)) OR (Health Counselling)) OR (Vision Screening)) OR (Health Prevention)) OR (Health Campaign)) OR (Health Protection)) OR (Health Behaviour)) OR (Ophthalmic Intervention)) OR (Programme)) OR (Outreach)		Search: (((Prevention of Blindness) OR (Reduce ocular morbidity)) OR (Increase the use of eye care services)) OR (Factors acting as barriers and facilitators to implementation of eye health promotion interventions) OR Improving eye health
	Search 1 result (#1) = 10,925	Search 2 result (#2) = 13,321,340		
	#1 AND #2 AND =8, 749			
