# Supplementary material for: Exploring barriers and facilitators, and their effectiveness in eye health promotion interventions: Protocol of a systematic review
Source: PLoS One. 2024 Sep 26;19(9):e0305904. doi: 10.1371/journal.pone.0305904 (PMC11426475; doi:10.1371/journal.pone.0305904)
Supplement: S4 Table — (PDF) [file pone.0305904.s011.pdf]

Author, Year	Type of Structured Intervention	Intervention Group		Control Group		SMD
		Mean (SD)	N	Mean (SD)	N	
