# Supplementary material for: Exploring barriers and facilitators, and their effectiveness in eye health promotion interventions: Protocol of a systematic review
Source: PLoS One. 2024 Sep 26;19(9):e0305904. doi: 10.1371/journal.pone.0305904 (PMC11426475; doi:10.1371/journal.pone.0305904)
Supplement: S5 Table — (PDF) [file pone.0305904.s012.pdf]

**Project Title:** Exploring barriers and facilitators, and their effectiveness in eye health promotion interventions: Protocol of a systematic review												
**Included references**	**Format**	**Database Searches [** *date run: / / ; date re-run: / /* **]**					**Supplementary Searches**					
		PubMed	***Web of Science***	***PsycINFO***	***CINAHL***	***MEDLINE***	***fcs***	***bcs***	***hs***	***wss***	***org***
Included ref 1											
Included ref 2											
Included ref 3											
No. of included refs											
No. of unique refs											
Yield											
No. of refs screened											
Sensitivity											
Precision											

Search-related information for the librarian or information specialist

No. of database searched =

Sum of yields =

No. of refs that underwent Title & Abstract screening =

No. of refs that underwent Full-text (FT) screening =

No. of included refs from database searching =

Total no. of included refs =

The summative metrics of effective searching

Overall sensitivity =

Overall precision =

NNR =

NNR FT =

NNS =

Codes

x = found from the search

y = in database; found when search strategy re-run

n = not in the database

z = in the database; not found using the search strategy

(red) = databases where searches re-run

NNR = number needed to read. 1/overall precision

NNR FT = number needed to read at FT to find one included reference

NNS = number needed to screen to find one reference to include for FT screening

Supplementary search codes

fcs = forwards citation search

bcs = backwards citation search

hs = hand search

wss = web site search

org = from contacting organisations

Format codes

jnl = journal article

ths = PhD thesis

Other codes

FT = full text
