# Supplementary material for: Exploring barriers and facilitators, and their effectiveness in eye health promotion interventions: Protocol of a systematic review
Source: PLoS One. 2024 Sep 26;19(9):e0305904. doi: 10.1371/journal.pone.0305904 (PMC11426475; doi:10.1371/journal.pone.0305904)
Supplement: S6 Table — (PDF) [file pone.0305904.s013.pdf]

Themes	Subthemes	Table
The Socio-Ecological Framework	**Multilevel conceptualization of the Socio-Ecological Framework**<br>1. **Intrapersonal Level**: The intrapersonal level of the socioecological framework considers the physical and cognitive characteristics of an individual as well as his or her prior experiences.<br>2. **Interpersonal Level**: The interpersonal level of the socioecological framework includes the community that surround the individual and can influence his or her safety.<br>3. **Organizational level**: The organizational level considers the structured communities to which groups of individuals belong.<br>4. **Environmental/Community level**: The environmental level can be described as the broader context surrounding an individual, including the cultural and physical environments.<br>5. **Public Policy**: Policies are enacted to safeguard individual and can be developed through the state association or state or national legislation.	Supplementary Table 6

Themes	Subthemes	Table
Ottawa charter for health promotion<br><br>(5 action areas of the Ottawa Charter for health promotion)	***Action areas of the Ottawa Charter for health promotion:***<br>1. Build Healthy Public Policy<br>2. Create Supportive Environments<br>3. Strengthen Community Actions<br>4. Develop Personal Skills<br>5. Reorient Health Services	Supplementary Table 6
